# Mortality Associated with Diabetes and Cardiovascular Disease in Older Women

**DOI:** 10.1371/journal.pone.0048818

**Published:** 2012-11-07

**Authors:** David Nanchen, Nicolas Rodondi, Jacques Cornuz, Teresa Hillier, Kristine E. Ensrud, Jane A. Cauley, Douglas C. Bauer, Study of Osteoporotic Fractures Research Group

**Affiliations:** 1 Department of Ambulatory Care and Community Medicine, University of Lausanne, Lausanne, Switzerland; 2 Department of Gerontology and Geriatrics, Leiden University Medical Center, Leiden, The Netherlands; 3 Department of General Internal Medicine, University of Bern, Bern, Switzerland; 4 Center for Health Research, Kaiser Permanente Northwest/Hawaii, Portland, Oregon, United States of America; 5 Center for Chronic Disease Outcomes Research, Veterans Affairs Medical Center, and Department of Medicine and Division of Epidemiology and Community Health, University of Minnesota, St. Paul, Minnesota, United States of America; 6 Department of Epidemiology, Graduate School of Public Health, University of Pittsburgh, Pittsburgh, Pennsylvania, United States of America; 7 Division of General Internal Medicine, Department of Medicine, University of California San Francisco, San Francisco, California, United States of America; Tehran University of Medical Sciences, Iran (Islamic Republic of)

## Abstract

**Background:**

Current guidelines for the prevention of cardiovascular disease (CVD) recommend diabetes as a CVD risk equivalent. However, reports that have examined the risk of diabetes in comparison to pre-existing CVD are lacking among older women. We aimed to assess whether diabetes was associated with a similar risk of total and cause-specific mortality as a history of CVD in older women.

**Methodology/Principal Findings:**

We studied 9218 women aged 68 years or older enrolled in a prospective cohort study (Study of Osteoporotic Fracture) during a mean follow-up period of 11.7 years and compared all-cause, cardiovascular and coronary heart disease mortality among 4 groups: non-diabetic women with and without existing CVD, diabetic women with and without existing CVD. Mean (SD) age of the participants was 75.2 (5.3) years, 3.5% reported diabetes and 6.8% reported existing CVD. During follow-up, 5117 women died with 36% from CVD. The multivariate adjusted risk of cardiovascular mortality was increased among both non-diabetic women with CVD (hazard ratio (HR) 2.32, 95% CI: 1.97–2.74, P<0.001) and diabetic women without CVD (HR 2.06, CI: 1.62–2.64, P<0.001) compared to non-diabetic women without existing CVD. All-cause, cardiovascular and coronary mortality of non-diabetic women with CVD were not significantly different from diabetic women without CVD.

**Conclusions/Significance:**

Older diabetic women without CVD have a similar risk of cardiovascular mortality compared to non-diabetic women with pre-existing CVD. The equivalence of diabetes and CVD seems to extend to older women, supporting current guidelines for cardiovascular prevention.

## Introduction

Cardiovascular disease (CVD) is the leading cause of death among older people [1]. Mortality rates of adults with diabetes have been shown to be similar to those with preexisting CVD [2] due to an increased risk of CVD among adults with diabetes [3]. Recent reviews reported a two-fold increased risk for CVD in adults with diabetes [4], [5]. Consequently, current international guidelines for the prevention of CVD define diabetes as a “CVD risk equivalent” and suggest similar management of cardiovascular risk factors in adults with diabetes and those with a history of CVD [6], [7]. However, several cohort studies have found conflicting results for CVD mortality comparing adults with diabetes and those with existing CVD [8], [9]. Differences in age, gender or the presence of other cardiovascular risk factors among populations studied might have accounted for these conflicting data [10], [11].

Few data exist comparing mortality risk in older diabetic women with those with CVD [12], [13]. Moreover, there might be large variations in CVD risk among older adults with diabetes, related to age at onset or shorter duration of diabetes [14]. Therefore, it remains unclear whether older diabetic women had a similar risk of mortality as compared to those with a history of CVD, as suggested by current guidelines for cardiovascular prevention.

The aim of this study was to determine whether older women with preexisting diabetes had the same risk of total and cardiovascular mortality as those with a history of CVD in the Study of Osteoporotic Fracture (SOF), a large cohort of older Caucasian women.

## Methods

### Ethics statement

Written informed consent was obtained from all women, and ethical approval was obtained by each center's institutional review board: Kaiser-Permanente Center for Health Research in Portland, Ore; University of Minnesota, Minneapolis; University of Maryland, Baltimore; and University of Pittsburgh, Pittsburgh, Pa.

### Study population

Between 1986 and 1988, 9704 Caucasian women (>99% Non-Hispanic White) aged 65 or older were enrolled in the SOF cohort, a multi-centre prospective observational study in four metropolitan areas in the United States (US): Baltimore, Monongahela Valley near Pittsburgh, Minneapolis and Portland [15], [16]. For this analysis, information was obtained at the third visit in 1991–1992 in 9218 participants aged 68 years or older who were alive or not lost to follow-up, because of missing information for history of CVD at the baseline visit in 1986–1988. Between the baseline and third visit (mean follow-up 3.5 years), the percentage of participant lost to follow-up was slightly lower for women with diabetes (0.44%) as compared to those without diabetes (0.67%). The primary purpose of SOF was to assess the risk factors for osteoporotic fractures. Participants were primarily recruited from population based mailings to community-based residents identified from voter-registration lists or memberships of large health maintenance organizations [15]. Women able to walk independently were included; those with bilateral hip replacement were excluded. African American women were also excluded because of their low incidence of osteoporotic fractures.

### Determinants and covariates

Diabetes status was based on self-report of physician diagnosis or use of anti-diabetic drugs, similar to previous reports from this cohort [17]. We further categorize diabetic women according to the duration of diabetes and defined late onset diabetes as having diabetes since 10 years or less, and long-term diabetes defined as having diabetes since more than 10 years, based on the self-reported date of diabetes onset. History of CVD was defined as coronary heart disease or stroke based on self-report of physician diagnoses. This self-reported information has been found to be effective for identifying cardiovascular disease in women [18]. Smoking status and years of education were assessed by interview. Smoking was classified as never, former and current. Physical activity was assessed by a modified version of the Harvard Alumni Questionnaire with information collected on exercise performed per week over the past year (kcal/wk) and by frequency of walking [19], as previously done in this cohort [20]. This questionnaire has been shown to be valid in women [21], [22]. Physical examination was performed with measurement of height, weight, waist girth and blood pressure according to standard protocols [23]. Hypertension was defined as a blood pressure ≥140/90 mmHg [24]. Daily current aspirin use was assessed by self-report. Body mass index (BMI) (kg/m^2^) was calculated from measured height and weight. Fasting total cholesterol, triglyceride and high-density lipoprotein (HDL) cholesterol levels were measured at the first visit on a random subsample at one SOF center (n = 958, 10.4%) [17], and were used in a sensitivity analysis.

### Outcomes

Follow up-contacts with ascertainment of vital status were carried out every 4 months and was 98% complete [16]. The coordinating center obtained copies of the original death certificates of all subjects who died. Cause of death was physician-adjudicated and based upon death certificates and discharge summaries (if available), with more than 95% of the underlying cause of death confirmed by the adjudicating physician [16]. According to ICD-9, cardiovascular mortality (402,404,410–414,426–445) was defined as mortality from atherosclerosis, stroke or coronary heart disease [24]. Coronary mortality (410–414) was defined as myocardial infarction or sudden death.

### Statistical analysis

Study participants were classified into 4 groups based on the presence or absence of diabetes or history of CVD: 1) no diabetes and no CVD ( = reference group), 2) no diabetes and history of CVD, 3) diabetes and no CVD, and 4) diabetes and history of CVD. Pairwise comparisons with the no diabetes and no CVD group were done with t-tests, chi-square or Mann-Whitney rank sum tests as appropriate. Kaplan-Meier curves and Cox proportional hazards models were used to assess the association between the 4 groups and mortality. We further examined the equivalence between late onset and long-term diabetes in comparison to the no diabetes and no CVD group (reference group), using Wald test from the Cox model. All models were adjusted for age and for potential confounders based on biological plausibility: smoking, physical activity, systolic blood pressure, body mass index and education. Further adjustment for lipid measurements was performed in a subsample of 952 participants with complete data available for cholesterol, HDL, triglycerides and low density lipoprotein (LDL) cholesterol [17]. We found similar estimates when using exercise performed per week instead of frequency of walking; therefore, we retained exercise performed per week in the final model. Results were reported as hazard ratios (HR) with 95% confidence intervals (CI).

## Results

The mean (SD) age of the 9218 women was 75.2 (5.3) years (Table 1). Six hundred twenty-four women (6.8%) reported a history of CVD without diabetes and 318 (3.5%) had diabetes without CVD. Women with diabetes were more likely to have hypertension, a larger body mass index, and did less physical activity than those without diabetes or CVD. Women with history of CVD only were older, more likely to be former smoker or aspirin user, to have hypertension, less years of school, and did less physical activity than those without diabetes or CVD.

**Table 1:** Characteristics of older women by presence or absence of diabetes and cardiovascular disease (CVD^a^) (N = 9218).

	No Diabetes		Diabetes	
	No CVD (n = 8181)	History of CVD (n = 624)	No CVD (n = 318)	History of CVD (n = 95)
Age, years	75.0±5.2	77.0±6.1**	74.6±4.8	76.9±5.8**
Smoking status^b^				
Never	60.8	56.9	65.9	61.0
Former	29.3	33.9	27.1	24.2
Current	9.9	9.2	6.9	14.7
Smoking, lifetime pack-years	10.3±19.5	13.0±22.6**	9.2±21.5	12.8±21.7
Systolic blood pressure, mmHg	141±19	144±20**	145±19**	148±20**
Diastolic blood pressure, mmHg	77±9	76±10	78±10	76±11
Hypertension	51.0	55.1*	59.8**	63.2*
Physical activity, total kcal/week^c^	1169 [504–2241]	880 [336–1866]**	837 [336–1774]**	605 [196–1374]**
Waist girth, cm	83.0±11.2	84.0±11.0*	91.3±12.8**	92.2±13.7**
Body mass index, kg/m^2^	26.3±4.6	26.1±4.7	28.6±5.3**	28.0±5.4**
Education, years	12.6±2.8	12.3±2.9**	12.3±2.6	12.2±2.9
Aspirin use	20.0	36.4**	16.3	31.6**

Values are mean ± standard deviation (SD) or percentages.

* p<0.05,

** p<0.005 for pairwise comparison with no diabetes and no CVD category.

p values were based on t-tests and chi-square tests for trend for multilevel categorical variables.

a CVD was defined as myocardial infarction or stroke.

b Former smoking was defined as at least 100 cigarettes for the all life. Chi-square tests for trend.

c Physical activity expressed as median [25%–75%] and Mann-Whitney rank sum test performed.

**Figure 1:**
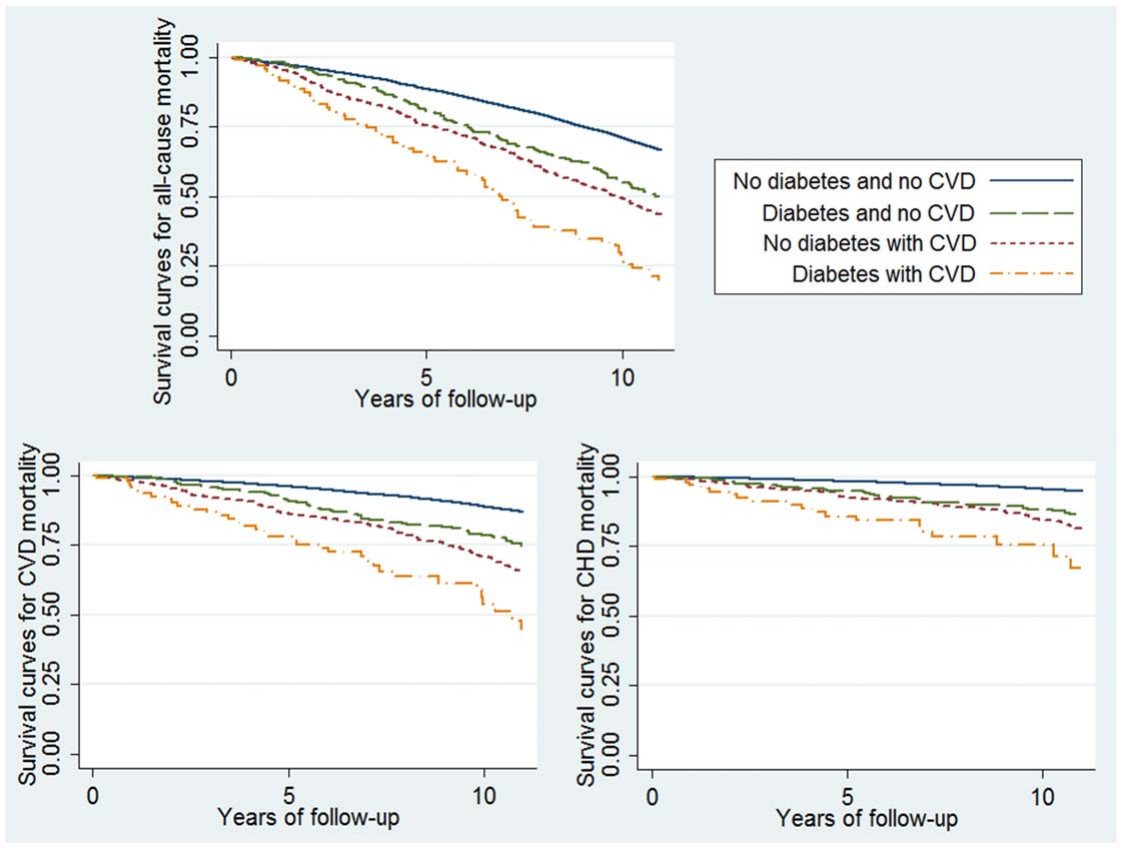
Survival curves according to the presence of diabetes or history of cardiovascular disease (CVD). a: Total mortality. b: Cardiovascular disease mortality. c: Coronary heart disease (CHD) mortality.

Mean study follow-up was 11.7 years to last visit or death. During follow-up, 5117 (55.5%) women died. Of these deaths, 1823 (35.6%) were attributed to CVD and 717 (14.0%) to coronary heart disease. Among the 8181 women without diabetes or CVD, 53% died and a third of these deaths were attributed to CVD. Among the 95 women with both diabetes and history of CVD, nearly 80% died and half of these deaths were attributed to CVD. During follow-up, unadjusted total, cardiovascular and coronary mortality was similar among women with diabetes and those with history of CVD (Figure 1).

Women with either diabetes or history of CVD or both conditions had a significantly higher total, cardiovascular and coronary mortality than women without diabetes nor CVD (Table 2). The association between diabetes or history of CVD with total and cardiovascular mortality remained in the multivariate adjusted models, as well as with further adjustment for aspirin use. Compared to non-diabetic women without history of CVD, cardiovascular mortality was elevated in both non-diabetic women with history of CVD with a multivariate adjusted HR of 2.32 (95% CI: 1.97–2.74, p<0.001), and in diabetic women without history of CVD with a multivariate adjusted HR of 2.06 (95% CI: 1.62–2.64, p<0.001). Cardiovascular mortality was highest among diabetic women with CVD (multivariate adjusted HR 3.93, 95% CI: 2.71–5.70, p<0.001). In the subsample of 958 (10.4%) older women with available lipid measurement, the association with total mortality persisted after further adjustment for LDL-cholesterol in women with diabetes (multivariate adjusted HR 1.50 (95% CI: 1.11–2.04, p = 0.009) or those with history of CVD (multivariate adjusted HR 1.78 (95% CI: 1.27–2.51, p = 0.001) compared to non-diabetic women without CVD.

**Table 2:** Incidence and hazard ratios (HR) with 95% confidence intervals (CI) for all-cause, cardiovascular and coronary mortality (N = 9218).

	No Diabetes		Diabetes	
	No CVD (n = 8181)	History of CVD (n = 624)	No CVD (n = 318)	History of CVD (n = 95)
**All-cause mortality**				
Deaths, N (%)	4371 (53.4)	451 (72.3)	220 (69.2)	75 (79.0)
Incidence, per 1000 person-years (95%CI)	44.5 (43.2–45.8)	78.0 (71.1–85.5)*	69.4 (60.8–79.2)*	113.9 (90.8–142.8)*
Age-adjusted HR (95%CI)	1.00	1.71 (1.55–1.89)*	1.86 (1.62–2.13)*	2.88 (2.29–3.62)*
Multivariate adjusted HR (95%CI)^a^	1.00	1.63 (1.45–1.83)*	1.96 (1.68–2.28)*	2.69 (2.06–3.51)*
Additionally adjusted for aspirin use (95% CI)	1.00	1.59 (1.41–1.78)*	2.00 (1.72–2.32)*	2.65 (2.02–3.46)*
**Cardiovascular mortality**				
Deaths, N (%)	1479 (18.1)	220 (35.3)	86 (27.0)	38 (40.0)
Incidence, per 1000 person-years (95%CI)	15.0 (14.3–15.8)	38.0 (33.3–43.4)*	27.1 (22.0–33.5)*	57.7 (42.0–79.3)*
Age-adjusted HR (95%CI)	1.00	2.40 (2.08–2.77)*	2.16 (1.74–2.68)*	4.18 (3.03–5.78)*
Multivariate adjusted HR (95%CI)^a^	1.00	2.32 (1.97–2.74)*	2.06 (1.62–2.64)*	3.93 (2.71–5.70)*
Additionally adjusted for aspirin use (95%CI)	1.00	2.24 (1.89–2.65)*	2.13 (1.66–2.72)*	3.86 (2.67–5.60)*
**Coronary mortality**				
Deaths, N (%)	547 (6.7)	105 (16.8)	45 (14.2)	20 (21.1)
Incidence, per 1000 person-years (95%CI)	5.6 (5.1–6.1)	18.1 (15.0–22.0)*	14.2 (10.6–19.0)*	30.4 (19.6–47.1)*
Age-adjusted HR (95%CI)	1.00	3.12 (2.52–3.86)*	2.97 (2.19–4.04)*	5.79 (3.70–9.08)*
Multivariate adjusted HR (95%CI)^a^	1.00	2.92 (2.28–3.73)*	2.76 (1.96–3.90)*	5.59 (3.38–9.25)*
Additionally adjusted for aspirin use (95%CI)	1.00	2.80 (2.19–3.59)*	2.84 (2.01–4.01)*	5.49 (3.32–9.08)*

Women with no diabetes and no history of CVD were used as the reference group.

* p<0.001 for comparison with the no diabetes and no CVD group. p values were based on log-rank tests for the rates of death and Wald test from the Cox model for other variables.

a Adjusted for age, smoking status, physical activity, systolic blood pressure, body mass index and education.

Abbreviations: CVD, cardiovascular disease.

We further examined the duration of diabetes and compared total and cause-specific mortality between 624 women without diabetes but history of CVD (reference group) and 191 women with late onset diabetes, as well as 126 women with long-term diabetes, both without history of CVD. Women with late onset diabetes and no history of CVD reported a first diagnosis of diabetes at age 60 years or later. The mean duration of diabetes was 5.8 years in the late onset diabetes group and 19.1 years in the long-term diabetes group. In age- and multivariate-adjusted models, diabetic women without CVD had similar cardiovascular and coronary mortality than non-diabetic women with history of CVD. Women with long-term diabetes seemed to have a higher mortality risk than women with late onset diabetes, but with large confidence intervals (Table 3).

**Table 3:** Comparison of hazard ratios (HR) with 95% confidence intervals (CI) for all-cause, cardiovascular and coronary mortality among non-diabetic women with history of CVD and diabetic women without CVD (N = 942).

	No Diabetes, History of CVD (n = 624)	Diabetes, No history of CVD (n = 318)	Late onset Diabetes^a^, No history of CVD (n = 191)	Long-term Diabetes^b^, No history of CVD (n = 126)
**All-cause mortality**				
Deaths, N (%)	451 (72.3)	220 (69.2)	128 (67.0)	92 (73.0)
Incidence, per 1000 person-years (95%CI)	78.0 (71.1–85.5)	69.4 (60.8–79.2)	65.6 (55.1–78.0)	76.4 (62.3–93.7)
Age-adjusted HR (95%CI)	1.00	1.03 (0.87–1.21)	0.98 (0.80–1.20)	1.10 (0.88–1.38)
Multivariate adjusted HR (95%CI)^c^	1.00	1.14 (0.94–1.38)	1.07 (0.85–1.36)	1.24 (0.96–1.60)
Additionally adjusted for aspirin use (95%CI)	1.00	1.24 (1.01–1.51)*	1.17 (0.92–1.50)	1.35 (1.04–1.76)*
**Cardiovascular mortality**				
Deaths, N (%)	220 (35.3)	86 (27.0)	47 (24.6)	39 (30.9)
Incidence, per 1000 person-years (95% CI)	38.0 (33.3–43.4)	27.1 (22.0–33.5)*	24.0 (18.1–32.0)*	32.4 (23.7–44.3)
Age-adjusted HR (95%CI)	1.00	0.83 (0.64–1.07)	0.75 (0.54–1.03)	0.96 (0.68–1.36)
Multivariate adjusted HR (95%CI)^c^	1.00	0.87 (0.65–1.16)	0.80 (0.56–1.16)	0.96 (0.65–1.43)
Additionally adjusted for aspirin use (95%CI)	1.00	0.91 (0.67–1.24)	0.85 (0.58–1.24)	1.02 (0.68–1.52)
**Coronary mortality**				
Deaths, N (%)	105 (16.8)	45 (14.1)	23 (12.4)	22 (17.5)
Incidence, per 1000 person-years (95%CI)	18.1 (15.0–22.0)	14.2 (10.6–19.0)	11.8 (7.8–17.7)	18.3 (12.0–27.7)
Age-adjusted HR (95%CI)	1.00	0.89 (0.62–1.27)	0.75 (0.47–1.18)	1.12 (0.70–1.77)
Multivariate adjusted HR (95%CI)^c^	1.00	0.92 (0.61–1.39)	0.76 (0.44–1.30)	1.14 (0.68–1.93)
Additionally adjusted for aspirin use (95%CI)	1.00	0.99 (0.64–1.53)	0.83 (0.48–1.43)	1.23 (0.72–2.11)

* p<0.05 for comparison with the no diabetes with CVD group.

Women with no diabetes and history of CVD were used as the reference group.

a Late onset diabetes defined as having diabetes since 10 years or less.

b Long-term diabetes defined as having diabetes since more than 10 years.

c Adjusted for age, smoking status, physical activity, systolic blood pressure, body mass index and education.

Abbreviations: CVD, cardiovascular disease.

## Discussion

In this large population-based study, the risk of cardiovascular and coronary mortality among older women with diabetes was similar to those with preexisting CVD. This increased mortality risk associated with diabetes was particularly apparent for longer duration of diabetes, and was independent of the presence of other cardiovascular risk factors.

Previous reviews of observational studies have summarized the cardiovascular risk associated with diabetes in comparison to those of adult with a history of CVD [25], [26]. However, most of the individual studies included in these meta-analyses have included middle-aged adults [8], [27], [28]. The heterogeneity of the populations studied in meta-analyses is also an issue, as a recent report has established epidemiological differences in the cardiovascular risk among men and women with diabetes [5]. For example, diabetes might confer a lower risk of coronary heart disease than previous myocardial infarction in middle-aged men [8], but a similar or higher risk in middle-aged women [27] and only few data exist for older women [13]. Therefore, our study provides important information to better assess the cardiovascular and coronary risk associated with diabetes in the population-based group of older women. In contrast to the limited power of traditional cardiovascular risk factors to predict CVD in older people [29], our results show that the presence of diabetes in older women - mean age of 75 years - was associated with total and cardiovascular mortality beyond demographics and other cardiovascular risk factors, and this association was as strong as the presence of pre-existing CVD.

In our study, the strong association of diabetes with total and cause-specific mortality might be explained by the long duration of exposure to diabetes. In middle-aged adults, the duration of diabetes plays a major role in the vascular risk [30]. In an observational study including women with a long duration of diabetes, diabetes was associated with a greater risk of coronary mortality than a history of coronary heart disease [12]. In older men, early-onset diabetes was associated with a higher risk of coronary heart disease than late onset diabetes [14]. When stratifying our results between older women with a shorter duration of diabetes (late onset defined as duration of less than 10 years) and those with long-term diabetes, the latter seemed to be associated with a worse prognosis. In addition, the nearly 12-year mean follow-up period of our study implies an exposure to many years of diabetes in both groups. Thus, in older diabetic women the duration of diabetes appears to be a key risk factor and further studies should evaluate its importance for better tailoring preventive intervention.

Our study has several limitations. We were not able to use the complete follow-up period of the SOF cohort which started in 1986–1988, because information on history of CVD was only available at the third visit in 1991–1992. However, there was no evidence for a selective loss of follow-up for women with diabetes as compared to the rest of the study population between the first and the third visit. Moreover, because of the long remaining 12-year follow-up period of this analysis and the very large number of deaths, we believe that our estimates are reliable. We may have also underestimated the number of women with diabetes and those with known CVD, because information was obtained by self-report of physician diagnosis or use of anti-diabetic drugs for women with diabetes. Many women could have had a subclinical diabetes or CVD. However, these underestimations would have likely decreased associations with mortality, which we still found to be statistically significant. Because cholesterol measurements were available for 10% of the whole population, models including adjustment with LDL-cholesterol were less precise, although estimates remained significant. Residual confounding was possible due to unrecorded diabetes therapy, such as rosiglitazone, a diabetes medication first approved in 2000 and associated with an increased incidence of CHD among women with diabetes [31]. However, over the bulk of follow-up time, this drug was not available. Baseline assessment of predictors was done in 1991–1992 and change in health behavior across recent decades might have influenced our results. However, diabetes has been shown to be associated with the same risk of cardiovascular mortality independently of the time-period [32]. Causes of deaths were based upon death certificates, but causes of death were confirmed by hospital discharge summaries and were adjudicated. These data have also a number of strengths: the large population of older women, the long follow-up period with a minimal loss to follow-up, and the very high number of events.

## Conclusions

In this large population-based study of older women, diabetes and CVD seem to have a similar impact on cardiovascular mortality and might need similar management as currently proposed by guidelines for cardiovascular prevention [6], [7]. Randomized controlled trials are needed to determine the intensity, the components and the safety of preventive measures that should be provided to older diabetic women, particularly if a history of CVD is additionally reported.

## References

[1] Lloyd-Jones D, Adams RJ, Brown TM, Carnethon M, Dai S, et al (2010) Heart disease and stroke statistics–2010 update: a report from the American Heart Association. Circulation 121: e46–e215. 20019324 10.1161/CIRCULATIONAHA.109.192667

[2] Haffner SM, Lehto S, Ronnemaa T, Pyorala K, Laakso M (1998) Mortality from coronary heart disease in subjects with type 2 diabetes and in nondiabetic subjects with and without prior myocardial infarction. N Engl J Med 339: 229–234. 9673301 10.1056/NEJM199807233390404

[3] Fox CS, Coady S, Sorlie PD, D'Agostino RB Sr, Pencina MJ, et al (2007) Increasing cardiovascular disease burden due to diabetes mellitus: the Framingham Heart Study. Circulation 115: 1544–1550. 17353438 10.1161/CIRCULATIONAHA.106.658948

[4] Sarwar N, Gao P, Seshasai SR, Gobin R, Kaptoge S, et al (2010) Diabetes mellitus, fasting blood glucose concentration, and risk of vascular disease: a collaborative meta-analysis of 102 prospective studies. Lancet 375: 2215–2222. 20609967 10.1016/S0140-6736(10)60484-9 PMC2904878

[5] Huxley R, Barzi F, Woodward M (2006) Excess risk of fatal coronary heart disease associated with diabetes in men and women: meta-analysis of 37 prospective cohort studies. BMJ 332: 73–78. 16371403 10.1136/bmj.38678.389583.7C PMC1326926

[6] Executive summary: Standards of medical care in diabetes (2012) Diabetes Care 35 Suppl 1: S4–S10. 22187471 10.2337/dc12-s004 PMC3632178

[7] Executive Summary of The Third Report of The National Cholesterol Education Program (NCEP) Expert Panel on Detection E, And Treatment of High Blood Cholesterol In Adults (Adult Treatment Panel III) (2001) JAMA 285: 2486–2497. 11368702 10.1001/jama.285.19.2486

[8] Evans JMM, Wang J, Morris AD (2002) Comparison of cardiovascular risk between patients with type 2 diabetes and those who had had a myocardial infarction: cross sectional and cohort studies. BMJ 324: 939. 11964337 10.1136/bmj.324.7343.939 PMC102325

[9] Lotufo PA, Gaziano JM, Chae CU, Ajani UA, Moreno-John G, et al (2001) Diabetes and all-cause and coronary heart disease mortality among US male physicians. Arch Intern Med 161: 242–247. 11176738 10.1001/archinte.161.2.242

[10] Gregg EW, Gu Q, Cheng YJ, Narayan KM, Cowie CC (2007) Mortality trends in men and women with diabetes, 1971 to 2000. Ann Intern Med 147: 149–155. 17576993 10.7326/0003-4819-147-3-200708070-00167

[11] Preis SR, Hwang SJ, Coady S, Pencina MJ, D'Agostino RB Sr, et al (2009) Trends in all-cause and cardiovascular disease mortality among women and men with and without diabetes mellitus in the Framingham Heart Study, 1950 to 2005. Circulation 119: 1728–1735. 19307472 10.1161/CIRCULATIONAHA.108.829176 PMC2789419

[12] Natarajan S, Liao Y, Sinha D, Cao G, McGee DL, et al (2005) Sex differences in the effect of diabetes duration on coronary heart disease mortality. Arch Intern Med 165: 430–435. 15738373 10.1001/archinte.165.4.430

[13] Pajunen P, Koukkunen H, Ketonen M, Jerkkola T, Immonen-Raiha P, et al (2005) Myocardial infarction in diabetic and non-diabetic persons with and without prior myocardial infarction: the FINAMI Study. Diabetologia 48: 2519–2524. 16247597 10.1007/s00125-005-0019-0

[14] Wannamethee SG, Shaper AG, Whincup PH, Lennon L, Sattar N (2011) Impact of diabetes on cardiovascular disease risk and all-cause mortality in older men: influence of age at onset, diabetes duration, and established and novel risk factors. Arch Intern Med 171: 404–410. 21403036 10.1001/archinternmed.2011.2

[15] Cummings SR, Black DM, Nevitt MC, Browner WS, Cauley JA, et al (1990) Appendicular bone density and age predict hip fracture in women. The Study of Osteoporotic Fractures Research Group. JAMA 263: 665–668. 2404146

[16] Kado DM, Browner WS, Palermo L, Nevitt MC, Genant HK, et al (1999) Vertebral fractures and mortality in older women: a prospective study. Study of Osteoporotic Fractures Research Group. Arch Intern Med 159: 1215–1220. 10371229 10.1001/archinte.159.11.1215

[17] Hillier TA, Rizzo JH, Pedula KL, Cauley JA, Schwartz AV, et al (2005) Increased mortality associated with the metabolic syndrome in older women with diabetes. Diabetes Care 28: 2258–2260. 16123504 10.2337/diacare.28.9.2258

[18] Heckbert SR, Kooperberg C, Safford MM, Psaty BM, Hsia J, et al (2004) Comparison of Self-Report, Hospital Discharge Codes, and Adjudication of Cardiovascular Events in the Women's Health Initiative. Am J Epidemiol 160: 1152–1158. 15583367 10.1093/aje/kwh314

[19] Paffenbarger RS Jr, Wing AL, Hyde RT (1978) Physical activity as an index of heart attack risk in college alumni. Am J Epidemiol 108: 161–175. 707484 10.1093/oxfordjournals.aje.a112608

[20] Gregg EW, Cauley JA, Seeley DG, Ensrud KE, Bauer DC (1998) Physical Activity and Osteoporotic Fracture Risk in Older Women. Ann Intern Med 129: 81–88. 9669990 10.7326/0003-4819-129-2-199807150-00002

[21] Wolf AM, Hunter DJ, Colditz GA, Manson JE, Stampfer MJ, et al (1994) Reproducibility and Validity of a Self-Administered Physical Activity Questionnaire. Int J Epidemiol 23: 991–999. 7860180 10.1093/ije/23.5.991

[22] Lee I-M, Rexrode KM, Cook NR, Manson JE, Buring JE (2001) Physical Activity and Coronary Heart Disease in Women. JAMA 285: 1447–1454. 11255420 10.1001/jama.285.11.1447

[23] Newman AB, Sutton-Tyrrell K, Rutan GH, Locher J, Kuller LH (1991) Lower extremity arterial disease in elderly subjects with systolic hypertension. J Clin Epidemiol 44: 15–20. 1986053 10.1016/0895-4356(91)90196-g

[24] Rodondi N, Taylor BC, Bauer DC, Lui LY, Vogt MT, et al (2007) Association between aortic calcification and total and cardiovascular mortality in older women. J Intern Med 261: 238–244. 17305646 10.1111/j.1365-2796.2007.01769.x

[25] Bulugahapitiya U, Siyambalapitiya S, Sithole J, Idris I (2009) Is diabetes a coronary risk equivalent? Systematic review and meta-analysis. Diabetic Medicine 26: 142–148. 19236616 10.1111/j.1464-5491.2008.02640.x

[26] Gonzalez-Clemente JM, Palma S, Arroyo J, Vilardell C, Caixas A, et al (2007) [Is diabetes mellitus a coronary heart disease equivalent? Results of a meta-analysis of prospective studies]. Rev Esp Cardiol 60: 1167–1176. 17996177

[27] Hu FB, Stampfer MJ, Solomon CG, Liu S, Willett WC, et al (2001) The impact of diabetes mellitus on mortality from all causes and coronary heart disease in women: 20 years of follow-up. Arch Intern Med 161: 1717–1723. 11485504 10.1001/archinte.161.14.1717

[28] Eberly LE, Cohen JD, Prineas R, Yang L (2003) Impact of incident diabetes and incident nonfatal cardiovascular disease on 18-year mortality: the multiple risk factor intervention trial experience. Diabetes Care 26: 848–854. 12610048 10.2337/diacare.26.3.848

[29] de Ruijter W, Westendorp RG, Assendelft WJ, den Elzen WP, de Craen AJ, et al (2009) Use of Framingham risk score and new biomarkers to predict cardiovascular mortality in older people: population based observational cohort study. BMJ 338: a3083. 19131384 10.1136/bmj.a3083 PMC2615548

[30] Fox CS, Sullivan L, D'Agostino RB, Wilson PW (2004) The significant effect of diabetes duration on coronary heart disease mortality: the Framingham Heart Study. Diabetes Care 27: 704–708. 14988289 10.2337/diacare.27.3.704

[31] Graham DJ, Ouellet-Hellstrom R, MaCurdy TE, Ali F, Sholley C, et al (2010) Risk of Acute Myocardial Infarction, Stroke, Heart Failure, and Death in Elderly Medicare Patients Treated With Rosiglitazone or Pioglitazone. JAMA 304: 411–418. 20584880 10.1001/jama.2010.920

[32] Berry JD, Dyer A, Cai X, Garside DB, Ning H, et al (2012) Lifetime risks of cardiovascular disease. N Engl J Med 366: 321–329. 22276822 10.1056/NEJMoa1012848 PMC3336876

